# Prevalence and genetic diversity of HCV among HIV-1 infected individuals living in Ahvaz, Iran

**DOI:** 10.1186/s12879-019-4052-x

**Published:** 2019-05-08

**Authors:** Ali Teimoori, Saeedeh Ebrahimi, Narges Keshtkar, Soheila Khaghani, Shokrollah Salmanzadeh, Shokouh Ghafari

**Affiliations:** 1 Infectious and Tropical Disease Research Center, Health Research Institute, Ahvaz Jundishapur University of Medical Sciences, Ahvaz, Iran; 2 Department of Virology, School of Medicine, Hamadan University of Medical Sciences, Hamadan, Iran; 3 Department of Health Services Management, School of Health, Ahvaz Jundishapur University of Medical Sciences, Ahvaz, Iran; 4 Infectious Diseases Research Center, Birjand University of Medical Sciences, Birjand, Iran

**Keywords:** HIV-1/HCV co-infection; HCV subtyping; prevalence of HCV, HIV, HCV, Co-infection, Genotypes

## Abstract

**Background:**

To explore the prevalence, transmission routes and genotypes distribution of HCV in HIV-1/HCV co-infected individuals in Ahvaz, Iran.

**Methods:**

The present cross-sectional study was conducted among HIV adult voluntary counseling and testing (VCT) clients, from September to November 2016. Reverse transcription (RT) nested PCR was performed to amplify the HCV core and 5′UTR regions from 90 HIV/HCV co-infected individuals. The PCR products were then sequenced for HCV subtyping. Genetic analysis was done by MEGA6 software.

**Results:**

The prevalence of HCV in HIV-1-infected individuals was 58.7%. Injection drug use (IDU) was the most common route (99.1%) of transmission, and most of the patients (97.8%) had a history of imprisonment. The HCV subtypes were identified as 1a (55.2%), 3a (35.8%), 3 h (4.5%), 1b (3%) and 4a (1.5%) respectively, HCV 1a and 3a subtypes were predominant.

**Conclusions:**

The diversity of HCV subtypes in HIV-1/HCV co-infected individuals in Ahvaz city was high, although two subtypes (1a and 3a) are predominant.

## Background

Hepatitis C virus (HCV) and Human Immunodeficiency virus (HIV) infections are major global public health problems [1]. Because of overlapping modes of transmission, HCV co-infection is common in HIV infected individuals but significantly varies depending on the mode of transmission from 10% among people with high risk sexual behavior to 90% among intravenous drug users [2].

Worldwide, an estimated 71 million people have chronic hepatitis C virus (HCV) infection and approximately 399,000 people die each year from hepatitis C-related liver diseases [3]. Likewise, about 2.3 million people of the estimated 36.9 million living with HIV globally have serological evidence of past or present HCV infection. HCV-associated liver disease represents a major cause of morbidity and mortality among HIV-infected patients [4].

HCV is a positive-stranded RNA virus belonging to the genus *hepacivirus* in the family of *Flaviviridae* [5]. HCV is classified into seven major genotypes; 67 confirmed and 20 provisional subtypes. HCV genotypes differ from each other at 30–35% of nucleotide sites, while at the subtypes level exhibits nucleotide divergence typically by &lt; 15% [6].

Recently, an eighth HCV genotype has been identified in four patients from Punjab, India [7].

It has already been established that the majority of HCV infections worldwide are caused by a few subtypes, specifically 1a, 1b, 2a, and 3a. These so-called “epidemic subtypes” were spread rapidly prior to the discovery of HCV by several routes. Many other HCV subtypes show an endemic pattern of transmission and are relatively rare that have circulated for long periods of time in a geographically restricted area [8].

HCV genotypes strongly influence the response to treatment. Therefore, understanding HCV genotypes is critical for appropriate treatment regimen. With conventional interferon alpha therapy, genotypes 1 and 4 are less responsive than genotypes 2 and 3. However, with new direct-acting antiviral (DAA) regimens, genotype 3 is most challenging to treat [9].

In Iran, HCV prevalence in the general population is estimated 0.3% [10] that is significantly lower than neighboring countries such as Afghanistan (1.1%), Pakistan (4.7%), Turkey (1.0–2.1%), Kuwait (0.8%) and Iraq (7.1%) [11]. According to the three conducted meta-analysis, the most frequent subtypes of HCV in Iran were 1a, 3a, and 1b respectively. This frequency differs in cities and provinces of Iran and neighboring countries [12–14]. In Iran, most of the studies have examined chronic HCV infection among hemodialysis and multiply transfused patients, especially those with hemophilia and thalassemia. In addition, a few studies recruited patients with a prison history, tattooing, unprotected high risk sexual behavior, and intravenous drug use. However, most studies did not report the genotypes and subtypes separately, based on population groups [13]. The present study was carried out in the Ahvaz, capital city of Khuzestan province in the southwest of the Iran, bordering Iraq and Arabian countries along the Persian Gulf. The Most important finding about HCV infection in Khuzestan has been acquired from seroprevalence studies that have been conducted in thalassemic and hemodialysis patients. The results from our previous study showed that CRF35_AD was only HIV-1 circulating subtype in this area [15]. There are no data available for the HCV prevalence and circulating subtypes in HIV infected persons. The aim of this study was to evaluate the prevalence and genetic diversity of HCV in HIV/HCV co-infected patients living in Ahvaz city, southwest of Iran.

## Methods

### Study design and population

This cross-sectional study was carried out in HIV/AIDS Voluntary Counselling and Testing (VCT) center in Ahvaz city, Iran. A registry of 1237 patients with HIV-1 infection was launched until September 2016 in this center. All records were retrieved and only 390 patients were referred to the center.

The sample inclusion criteria were adult patients, who had at least one appointment in the previous 12 months, co-infected by HCV. Finally, 229 HIV/HCV co-infected patients were followed up. The exclusion criterion was current or previous therapy with DAA.

All HIV/HCV co-infected patients invited to participate in this study from September to November 2016 (3 month). A majority of co-infected persons claimed to be under DAA treatment or refused to participate in the survey. Finally, only 90 eligible patients were enrolled. The written informed consent was obtained prior to data collection in accordance with the Helsinki Declaration and patients were asked to fill the social demographic questionnaire. About 5 mL of peripheral blood was taken from each patient and collected in K2-EDTA vacutainer tubes. The plasma was separated by centrifugation at 400 g for 10 min and stored at − 80 °C.

### RNA extraction and genotyping

Viral RNA was extracted from 140 μL of plasma by using the QIAamp viral RNA mini kit (Qiagen, Germany), according to the manufacturer’s instructions. The yield and purity of the RNA was determined using NanoDrop (Thermo Scientific, USA) and the samples were stored at − 80 C for further use. To determine the genotype of HCV, the partial region of core gene were amplified using nested polymerase chain reaction (PCR) with two sets of modified primers, as described by ohno, et al. [16] (Table 1). cDNA synthesis and the first round of PCR were performed using QIAGEN one-step RT-PCR Kit (Qiagen, Germany). I-Taq Maxime PCR Premix (iNtRON Biotechnology, Korea) was used for further amplification by nested PCR. All PCR products were purified using the QIAquick Gel Extraction kit (Qiagen, Germany) and were sequenced in both directions using a Big Dye Terminator v3.1 Cycle Sequencing kit (Applied Biosystems, USA) and the ABI PRISM 3730xl DNA Analyzer. In cases of failure of core region amplifying, 5′UTR (5′untranslated) region was amplified by using nested PCR with two sets of previously described primers [17] (Table 1).

**Table 1:** The sequences of used primers

Name	Sequence	Nucleotides	Orientation	Usage
Sc2	GGGAGGTCTCGTAGACCGTGCACCATG	318 → 344	Core/outer	Forward
Ac2	GAGCGGGATATACCCCATGAG(A/G)TCGGC	758 → 732	Core/outer	Reverse
S7	AGACCGTGCACCATGAGCAC	330 → 349	Core/inner	Forward
584	CCCATGAGGTCGGC(A/G)AAGC	749 → 730	Core/inner	Reverse
BKP-7	CACTCCCCTGTGAGGAACTACTGTC	38 → 62	5’UTR/outer	Forward
BKP-8	ATGGTGCACGGTCTACGAGACCTCC	343 → 319	5’UTR/outer	Reverse
BKP-9	TTCACGCAGAAAGCGTCTAGCCATG	63 → 87	5’UTR/inner	Forward
BKP-10	GCGCACTCGCAAGCACCCTATCAGG	314 → 292	5’UTR/inner	Reverse

All sequence fragments in core and 5′UTR regions were assembled using DNA Sequence Assembler v4 (2013). The assembled sequences were aligned with HCV reference sequences using the CLUSTALW Multiple alignments and manual editing in BioEdit software 7.2.5. The HCV reference sequences retrieved from LOS ALAMOS HCV database (http://www.hiv.lanl.gov).

Moreover, All nucleotide sequences were screened using the BLAST search tool (http://blast.ncbi.nlm.nih.gov/Blast.cgi) to search for sequence similarities to previously reported sequences in the database. The alignments were used to construct the phylogenetic tree by a neighbor-joining (NJ) method in MEGA6 software [18] using the Kimura 2-parameter (K2P) model of evolutionary [19] distance with pairwise-deletion and 1000 bootstrap replicates .

### Virological and immunological tests

The HCV antibody was determined using the third-generation enzyme immunoassays (DIA.PRO Diagnostic, Bioprobes Srl, Milan, Italy). HIV-1 infection was diagnosed by using rapid HIV test followed by both ELISA and Western blot confirmatory tests, in accordance with the HIV national algorithms in Iran. CD4 cell counts were determined using the Becton Dickinson (BD) FASCount system (Becton, Dickinson, USA).

## Results

In this study we analyzed all records obtained from VCT center in Ahvaz city and found that the prevalence of HCV in HIV-1-infected individuals was 58.7%. Injection drug use (IDU) was the most common route (99.1%) of transmission, and most of the patients (97.8%) had a history of imprisonment. A total of 90 HCV/HIV-1 co-infected patients, including 88 (97.8%) men and 2 (2.2%) women were consecutively enrolled during sampling time (3 month) from VCT center in Ahvaz, Khuzestan.

The median age of participants was 31 years (range: 18–43 years) and the median CD4+ T-cell count was 302 cells/μl (range: 22 to 832 cells/μl). Most patients were single (53.4%) and unemployed (60%). Educational level varied from illiterate to diploma. HCV/HIV transmission risks were injection drug use (98.9%), heterosexual contact (74.5%), male-to-male (MSM) sexual contact (21.1%) and tattooing (80%). The overall prevalence of high-risk sexual behaviors in all participants was 95.6%. The majority of cases (87.8%) were under HAART treatment. The median alanine aminotransferase (ALT) and aspartate aminotransferase (AST) were 68 U/L and 56 U/L respectively. Demographic and laboratory characteristics of participants are shown in Table 2.

**Table 2:** Demographic, and laboratory characteristics of HIV-HCV coinfection status groups (Total population and enrolled participants)

Characteristic	Total population (*n* = 229)	Participants(*n* = 90)
Median age, years	32	31
Gender		
Male	225 (98.2)	88 (97.8)
Female	4 (1.8)	2 (2.2)
Marital status		
Single	87 (38)	48 (53.4)
Married	72 (31.5)	30 (33.4)
Divorced/separated	69 (30.1)	11 (12)
Widow	1 (0.4)	1 (1.2)
Occupation		
Unemployed	147 (64.2)	54 (60)
Self-employed	82 (35.8)	36 (40)
Employed	0 (0)	0 (0)
Literacy		
Illiterate	11 (4.8)	2 (2.2)
Primary school	134 (58.5)	59 (65.6)
Secondary school	82 (35.8)	27 (30)
Diploma	2 (0.9)	2 (2.2)
University degree	0 (0)	0 (0)
Risk factors		
IDU*	227 (99.1)	89 (98.9)
High risk sexual behavior	222 (96.9)	86 (95.6)
Heterosexual	185 (80.8)	67 (74.5)
Male-to-male sexual contact	37 (16.2)	19 (21.1)
Tattoo	196 (85.6)	72 (80)
Prison record	224 (97.8)	88 (98)
CD4 cell count-median cells/μl	244	302
HAART**	206 (90)	79 (87.8)
Liver Blood Enzymes(units/l)		
ALT (SGPT)***	79	68
AST (SGOT)****	63	56

* Injection drug use **** Aspartate aminotransferase

** Highly active antiretroviral therapy

*** Alanine aminotransferase

Among 90 HIV-HCV co-infected patients, according to the anti-HCV test, the infection was confirmed based on a positive HCV core RNA for 64 (71.2%) participants. Also, 5′UTR RNA was detected in 3 samples of 26 samples that failed to amplify. In total, the presence of HCV*-*RNA was confirmed in 67 (74.5%) samples.

**Fig. 1:**
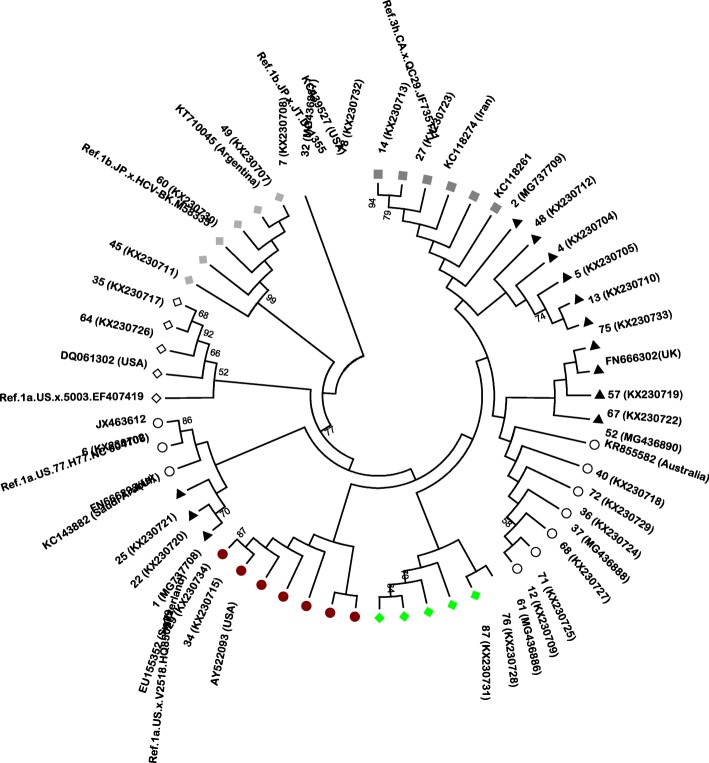
Phylogenetic analysis of partial core sequences of HCV subtypes 1a, and 1b isolated from patients co-infected with HIV/HCV in Ahvaz, Iran. The sequences found in this study are shown with number. A panel of reference strains retrieved from LOS ALAMOS HCV database identified by their accession number. The origins of reference strains are indicated. Data regarding the geographic origin of HCV reference sequences of Iranian origin are also indicated when available in the GenBank database. Tree were constructed with a neighbor-joining (NJ) method in the MEGA6 software using the Kimura 2-parameter (K2P) model with pairwise-deletion and 1000 bootstrap replicates. Bootstrap values over 50% are shown

**Fig. 2:**
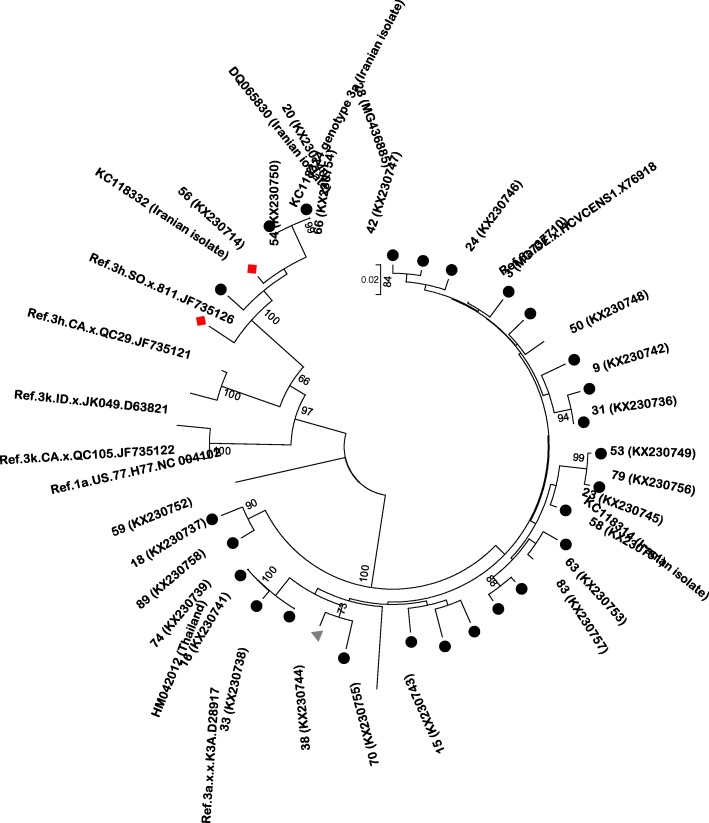
Phylogenetic analysis of partial core sequences of HCV subtypes 3a, 3b, and 3 h isolated from patients co-infected with HIV/HCV in Ahvaz, Iran. The sequences found in this study are shown with number. A panel of reference strains retrieved from LOS ALAMOS HCV database identified by their accession number. The origins of reference strains are indicated. Data regarding the geographic origin of HCV reference sequences of Iranian origin are also indicated when available in the GenBank database. Tree were constructed with a neighbor-joining (NJ) method in the MEGA6 software using the Kimura 2-parameter (K2P) model with pairwise-deletion and 1000 bootstrap replicates. Bootstrap values over 50% are shown

None of the included patients had previously been treated for HCV. Phylogenetic analysis of the core region sequences of 64 samples showed that 35 samples were infected with genotype 1a, 23 with 3a, three with 3 h, two with 1b and one with 4a (Table 3). As mentioned above, from cases of failure, 5′UTR RNA was amplified and detected in 3 cases. Two of them was genotype1a and the remaining was 3a. Totally, the 1a HCV genotype was predominant (55.2%), followed by the 3a genotype (35.8%). Subtypes 4 and 5′UTR were not shown here. (Figs. 1 and 2).

**Table 3:** Frequency of HCV subtypes among patients

HCV subtype	Frequency (%)
1a	37 (55.2)
1b	2 (3)
3a	24 (35.8)
3 h	3 (4.5)
4a	1 (1.5)
Total	67 (100)

## Discussion

In this study, we found that the seroprevalence rate of HCV in HIV-infected people was 58.7% (229/390). In addition, the majority of participants were male (97.8%); due to the fact that the majority of HIV cases in Iran were male. In Iran, the prevalence of hepatitis C virus varies widely among different population groups. It is estimated from 0.3% in the general populations until 32.1% among high risk populations [10]. The findings of this study revealed that the prevalence of HCV infection among HIV infected individuals was significantly higher than the general population. This occurred because the majority of co-infected individuals were injection drug users (IDUs). Our results were consistent with the published data, suggesting that HCV co-infection rates are very high among HIV-infected individuals who use injection drugs [20–22]. In addition, various studies in Iran showed that more than 42.2–85% of HIV-positive IDUs were co-infected with HCV [23–25].

In other hands, the majority (97.8%) of HIV/HCV co-infected patients were previously incarcerated, and some of them had re-incarceration records. Incarceration is a significant risk factor for acquisition of HIV and HCV globally. There is strong evidence that incarceration is associated with increased HIV and HCV acquisition risk among injection drug users [22].

Co-infection with HIV accelerates the progression of HCV-related fibrosis and results in a more aggressive course of liver disease [26]. For this reason, treatment of HCV in this patient population should have a high priority. HCV genotype significantly affects treatment outcome; therefore, genotyping is more predictive for therapy response [27].

Currently, the gold standard for identifying different HCV genotypes is sequence analysis of specific regions (NS5, core, E1 and 5′UTR) followed by sequence alignment with reference sequences (the consensus sequences in GenBank or the Los Alamos HCV database) and phylogenetic analysis [28, 29]. The present study was performed on 90 individuals who were infected with HIV and have anti-HCV Abs, indicative of virus exposure, to evaluate the presence of HCV infection and HCV genotype. We identified five HCV subtypes in HIV-infected people in Ahvaz, including 1a, 1b, 3a, 3 h, and 4a (Table 3).

Our results in terms of genetic diversity were inconsistent with some previous studies conducted in this area [30, 31]. The cause of this difference can be due to the two factors; (a) methodology, and (b) study populations. None of these studies used sequencing methods for genotyping and examined haemophilia patients and blood donors.

Despite these differences, we found that subtype 1a (55.2%) was the most predominant, followed by subtype 3a (35.8%), which was consistent with other reports in different population groups in this area [31, 32]. It seems that, almost 10 years after the first study in this area, subtypes 1a have remained predominant regardless of the population studied.

The major limitation of this study was the nature of cross-sectional study and small sample size, therefore, participants in this study may not be representative of HIV-1/HCV co-infected individuals from the region. Because of the small sample size, causal relationship between CD4+ T cell counts and different HCV subtypes could not be tested. Additionally, most participants received HAART and we could not find the relationship between liver enzyme (ALT, AST) levels and HCV subtypes.

## Conclusions

The present study is the first report on the prevalence and genetic diversity of hepatitis C virus in HIV-infected individuals in southwest Iran. These results suggest that HIV-infected patients are exposed to HCV at a higher rate than the general population. Intravenous drug use plays an important role in the transmission of this virus. Furthermore, this data shows that there are two major circulating subtypes of HCV in this area: subtypes 1a and 3a.

### Sequence accession numbers

The Genbank accession numbers for the sequences reported in this paper are KX230704–58, KX258961, and MG737708–11 for the partial core region, and MG436880–83 for the partial 5′UTR region.

## Abbreviations

ALT: Alanine aminotransferase
AST: Aspartate aminotransferase
DAA: Direct-acting antiviral
HAART: Highly active antiretroviral therapy
HCV: Hepatitis C virus
HIV: Human immudeficiency virus
IDU: Injection drug use
PCR: Polymerase chain reaction
VCT: Voluntary counseling and testing
